# Anti-NLRP3 Inflammasome Natural Compounds: An Update

**DOI:** 10.3390/biomedicines9020136

**Published:** 2021-02-01

**Authors:** Baolong Liu, Jiujiu Yu

**Affiliations:** Department of Nutrition and Health Sciences, University of Nebraska-Lincoln, Lincoln, NE 68583, USA; baolong@huskers.unl.edu

**Keywords:** NLRP3 inflammasome, natural compounds, inflammatory diseases, medicinal herbs

## Abstract

The nucleotide-binding domain and leucine-rich repeat related (NLR) family, pyrin domain containing 3 (NLRP3) inflammasome is a multimeric protein complex that recognizes various danger or stress signals from pathogens, the host, and the environment, leading to activation of caspase-1 and inducing inflammatory responses. This pro-inflammatory protein complex plays critical roles in pathogenesis of a wide range of diseases including neurodegenerative diseases, autoinflammatory diseases, and metabolic disorders. Therefore, intensive efforts have been devoted to understanding its activation mechanisms and to searching for its specific inhibitors. Approximately forty natural compounds with anti-NLRP3 inflammasome properties have been identified. Here, we provide an update about new natural compounds that have been identified within the last three years to inhibit the NLRP3 inflammasome and offer an overview of the underlying molecular mechanisms of their anti-NLRP3 inflammasome activities.

## 1. Introduction

The nucleotide-binding domain and leucine-rich repeat related (NLR) family, pyrin domain containing 3 (NLRP3) inflammasome is a cytosolic protein complex in the innate immune cells. It is composed of NLRP3, apoptotic speck protein containing a caspase recruitment domain (ASC), and caspase-1 [1,2]. In response to a variety of pathogen-associated molecular patterns (PAMPs) and danger-associated molecular patterns (DAMPs), the sensor NLRP3 recruits the adaptor ASC, which further recruits the effector caspase-1 to form the inflammasome complex. Caspase-1 autocleaves itself to generate the active enzyme, which cleaves pro-interleukin (IL)-1β and pro-IL-18 to generate the mature cytokines IL-1β and IL-18. Active caspase-1 also cleaves gasdermin D to induce pyroptotic cell death to amplify the inflammatory responses [3,4].

The NLRP3 inflammasome has become an attractive therapeutic target because growing evidence has shown that its inappropriate activation is involved in the pathogenesis of many complex diseases, such as Alzheimer’s disease, type 2 diabetes, and gout [5,6]. In clinical practice, protein-based therapies that block the action of IL-1β have been tested in patients. For example, injection of anakinra, a recombinant human IL-1 receptor antagonist protein, in diabetic patients suppressed systemic inflammation, lowered plasma glucose, and improved β-cell secretory function [7,8]. Rilonacept, a soluble decoy IL-1 receptor, reduced inflammatory markers, pain, and swelling in gout patients in clinical trials [9]. While these protein-based therapies are promising, they are costly, require frequent injection, and block only one downstream pathway of the NLRP3 inflammasome. Therefore, it is highly desirable to identify other potential therapeutic interventions that target the NLRP3 inflammasome.

Remarkably, many natural compounds or nutrient molecules have been shown to suppress activation of the NLRP3 inflammasome. Two recent reviews [10,11] provide a comprehensive overview of these natural products. In this review, we will (1) introduce the background information about NLRP3 inflammasome activation and its involvement in diseases, and (2) provide an update of natural compounds with anti-NLRP3 inflammasome functions that have been identified within the last three years.

## 2. NLRP3 Inflammasome and Its Involvement in Diseases

### 2.1. Activation of the NLRP3 Inflammasome

**Figure 1:**
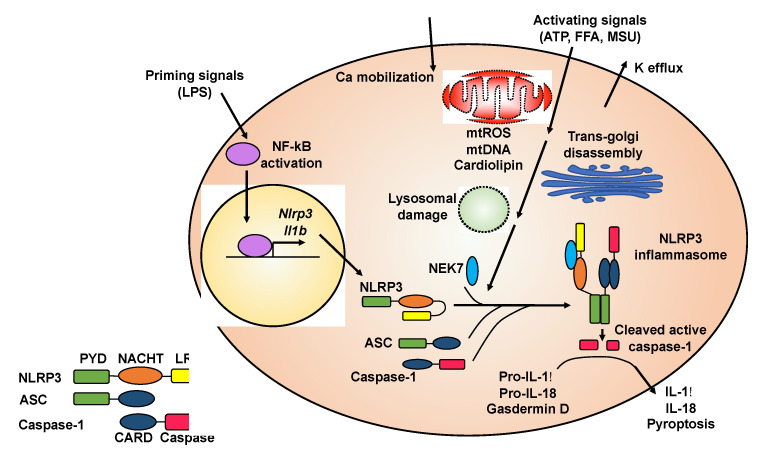
Molecular mechanisms of NLRP3 inflammasome activation. The NLRP3 inflammasome is activated through an initial priming step followed by an activating step. The priming signals, such as lipopolysaccharide (LPS), activate NF-κB signaling, which induces transcription of the *Nlrp3* and *Il1b* genes. The activating signals, such as ATP, nigericin, free fatty acids (FFAs), monosodium urate (MSU) or cholesterol crystals, can trigger a series of cellular events, which typically include potassium efflux, calcium mobilization, mitochondrial (mt) damage (leading to release of reactive oxygen species (ROS), DNA, and cardiolipin), trans-Golgi disassembly, and lysosomal rupture. These upstream cellular events converge on the NLRP3 protein, causing its conformational change and leading to oligomerization of NLRP3 with the assistance of NEK7 protein. Oligomerized NLRP3 recruits ASC, which further recruits caspase-1, to form the NLRP3 inflammasome complex. Caspase-1 autocleaves itself to generate the active enzyme, which cleaves pro-IL-1β and pro-IL-18 to release mature cytokines. Active caspase-1 also cleaves gasdermin D to induce pyroptosis. Both cytokine release and pyropototic cell death trigger inflammation.

The molecular mechanisms of NLRP3 inflammasome activation have been reviewed in depth [12,13,14,15,16]. Here, we summarize general principles of NLRP3 inflammasome activation (Figure 1) to provide background knowledge about how natural compounds target this key pro-inflammatory protein complex.

Although the precise molecular mechanisms of NLRP3 inflammasome activation are not fully understood, it is generally accepted that NLRP3 inflammasome activation requires an initial priming step followed by an activation step (Figure 1) [12,13]. A priming signal, such as lipopolysaccharide (LPS), activates nuclear factor kappa-light-chain-enhancer of activated B cells (NF-κB) signaling, leading to increased expression of the *Il1b* and *Nlrp3* genes. The priming signal also induces the deubiquitination of NLRP3 protein, which is a prerequisite for optimal activation of the NLRP3 inflammasome [17,18,19]. Activating signals for the NLRP3 inflammasome include a variety of molecules with diverse and un-related chemical structures. For example, ATP, nigericin, alum, free fatty acids (FFAs), cholesterol crystals, or monosodium urate (MSU) crystals can serve as an activating signal to promote the assembly and formation of the NLRP3 inflammasome [12,13].

The exact signaling cascade from activating signals or NLRP3 agonists to inflammasome assembly is still not clear. Some upstream cellular events that have been implicated in NLRP3 inflammasome formation include potassium efflux, calcium mobilization, production of mitochondrial reactive oxygen species (ROS), release of mitochondrial DNA, cytosolic exposure of mitochondrial inner member lipid cardiolipin, cellular translocation of inflammasome subunits, lysosomal destabilization, and trans-Golgi disassembly (Figure 1) [2,12,14]. However, some of these upstream cellular events are agonist specific. For example, lysosomal destabilization is often observed when crystal agonists or protein aggregates are used to activate the NLRP3 inflammasome [20,21,22]. In general, it seems that all NLRP3 agonists trigger cellular stress, which could be sensed by the NLRP3 protein, leading to NLRP3 inflammasome assembly and activation.

The NLRP3 protein contains an N-terminal pyrin domain (PYD), a central nucleotide-binding and oligomerization domain (NACHT), and a C-terminal leucine-rich repeat domain (LRR) (Figure 1) [6]. In the absence of activating signals, LRR internally interacts with NACHT, which causes the NLRP3 protein to form a closed structure. The activating signal triggers a conformational transition of NLRP3 proteins, leading to the oligomerization of NLRP3 through their NACHT domains. Recently, it has been shown that adjacent NLRP3 proteins are bridged through never in mitosis gene a (NIMA)-related kinase 7 (NEK7) to facilitate oligomerization [23]. Oligomerized NLRP3 forms a platform to recruit ASC through PYD–PYD interaction. ASC contains an N-terminal PYD and a C-terminal caspase activation and recruitment domain (CARD). After PYD of ASC interacts with PYD of NLRP3, the CARD of ASC further recruits caspase-1 through CARD–CARD interaction, since caspase-1 contains an N-terminal CARD and a C-terminal caspase domain. The recruitment of caspase-1 in the inflammasome complex triggers its autocleavage to generate the active enzyme [1,2]. Because both PYD and CARD form filaments, the resulting NLRP3 inflammasome is a highly polymerized and filamentous protein complex with high molecular mass [24].

### 2.2. Involvement of the NLRP3 Inflammasome in Diseases

While proper innate immune responses protect against insults, aberrant activation of the NLRP3 inflammasome causes or contributes to the onset and progress of various diseases. Here, we summarize the involvement of the NLRP3 inflammasome in a wide range of diseases to highlight the pathophysiological roles of this protein complex.

Cryopyrin-associated periodic syndromes (CAPS). Dominant missense mutations in the *NLRP3* gene in humans result in a hyperactive NLRP3 inflammasome and overproduction of inflammatory cytokines, leading to CAPS, a group of autoinflammatory diseases that include familial cold autoinflammatory syndrome (FCAS), Muckle–Wells syndrome (MWS), and neonatal onset multisystem inflammatory disease (NOMID) [25,26]. CAPS patients experience varying degrees of systemic inflammation. FCAS, at the mild end, is characterized by cold-induced inflammatory episodes including recurrent hives, joint pain, and fever. MWS, of intermediate severity, manifests recurrent inflammatory attacks often accompanied by progressive hearing loss and sometimes amyloidosis in the kidney. The most severe NOMID manifests persistent inflammation from birth, as well as severe tissue damage mainly affecting the nervous system, skin, and joints. Mice carrying specific CAPS-associated mutations in the *Nlrp3* gene demonstrate inflammatory symptoms similar to those seen in CAPS patients [27,28].

Gout. NLRP3 inflammasome activation is implicated in the etiology of gout. Gout is a common form of inflammatory arthritis characterized by severe and intense pain, swelling, redness, and heat often occurring at the big toe joint [29]. Gouty arthritis is caused by the deposition of MSU crystals in joints, which—remarkably—activate the NLRP3 inflammasome [30]. Deletion of the *Nlrp3*, *Pycard* (*Asc*), or *Casp1* gene in mice leads to reduced articular inflammation in response to MSU injection in joints [31].

Multiple sclerosis (MS). MS is an autoimmune inflammatory disease of the brain and spinal cord. In MS, inflammation occurs in the myelin layer around the nerves and causes scars or lesions in the myelin, disrupting communication in the nervous system [32]. The most common symptoms of MS are fatigue and difficulty walking, but the symptoms, severity, and duration can vary widely among patients. Expression of the *CASP1* and *IL18* genes increased in the peripheral blood mononuclear cells (PBMCs) of MS patients [33]. Expression of the *Nlrp3* gene increased in the spinal cord during the progression of experimental autoimmune encephalomyelitis (EAE), an animal model of MS, and deletion of the *Nlrp3*, *Casp1*, *Pycard*, or *Il1b* genes in mice led to resistance to EAE [34,35,36].

Alzheimer’s disease (AD). AD is a progressive brain disorder caused by gradual death of neuronal cells in the hippocampus and cerebral neocortex [37]. The symptoms of AD include memory loss, problems with communication, reasoning, and thinking, and eventually loss of ability to function independently. Expression of the *NLRP3* and *CASP1* genes was enhanced in the brain of AD patients [38,39]. β-amyloid deposition in the brain is a key factor that drives the development of AD [40], and remarkably, β-amyloid aggregates activate the NLRP3 inflammasome [22]. Knockout of the *Nlrp3* or *Casp1* genes reduced neuronal inflammation and improved cognitive function in the AD mouse model [38].

Parkinson’s disease (PD). PD is a neurodegenerative disease caused by gradual death of dopamine-producing neurons [41]. Very often, PD patients experience tremor, stiffness, and difficulty with walking, balance, and coordination. In mice, PD can be induced by administration of 1-methyl-4-phenyl-1,2,3,6-tetrahydropyridine (MPTP), a chemical leading to the loss of dopamine-producing neurons. *Nlrp3*^−/^^−^ mice were resistant to development of PD after MPTP treatment [42], suggesting the involvement of the NLRP3 inflammasome in PD etiology. In addition, aggregated α-synuclein, which is considered a key element in the pathogenesis of PD, has been shown to activate the NLRP3 inflammasome [43].

Obesity. Obesity is a chronic progressive disease that leads to development of type 2 diabetes, heart disease, and stroke, all of which are among the top 10 leading causes of death in the United States [44,45]. In obesity, the level of circulating LPS, which acts as the priming signal for the NLRP3 inflammasome [5,14,15], from gut microbiota is significantly enhanced [46,47]. Nutrient excess in obesity leads to an accumulation of metabolic stress molecules, such as FFAs, cholesterol crystals, glucose, or amyloid polypeptides [5,14,15], which serve as the second activation signal to promote the assembly and activation of the NLRP3 inflammasome. Expression of the *NLRP3*, *ASC*, and *IL1B* genes has been shown to increase in PBMCs, subcutaneous adipose tissue, and visceral adipose tissue in obese humans compared to lean subjects [48,49]. When the adipose tissue or monocyte-derived macrophages were cultured ex vivo, the levels of secreted cytokines IL-1β and IL-18 were much higher in obese samples compared to lean samples. Cytokine levels were tightly associated with an increased number of adipose tissue macrophages, increased plasma glucose and insulin levels, and higher metabolic syndrome scores in obese patients [48,50,51]. Genetic deletion of the *Nlrp3*, *Pycard*, or *Casp1* genes in diet-induced obese mice improved glucose tolerance and insulin sensitivity, accompanied by decreased inflammatory cytokines in the serum and reduced expression of inflammatory genes in metabolic tissues, including adipose tissue and liver [52,53,54].

Atherosclerosis. Atherosclerosis refers to the buildup of plaque, composed of cholesterol crystals, immune cells, and other substances, on the arterial wall [55]. Over time, the plaque hardens and narrows the arteries and eventually leads to severe conditions such as heart attack or stroke. In aortas of patients with coronary atherosclerosis, the levels of the NLRP3 protein were much higher compared to individuals without atherosclerosis [56]. Cholesterol crystals activate the NLRP3 inflammasome [21]. The low-density lipoprotein receptor deficiency (*Ldlr*^−/^^−^) mouse is a widely used atherosclerosis model [57]. When these mice were transplanted with bone marrow from *Nlrp3*^−/^^−^, *Pycard*^−/^^−^, *Il1b*^−/^^−^ or *Casp1*^−/^^−^ mice, they had decreased sizes of aortic lesions [21,58]. Atherosclerosis was ameliorated upon deletion of the *Casp1* gene in apolipoprotein E deficiency (*ApoE*^−/^^−^) mice (also a widely used atherosclerosis mouse model) [59]. However, another study showed that atherosclerosis in *ApoE*^−/^^−^ mice is not dependent on the NLRP3 inflammasome [60]. The exact reasons for this discrepancy are not clear.

Type 2 diabetes. The key symptom of type 2 diabetes is an elevated glucose level in the circulatory system, which is accompanied by insulin resistance, pancreas failure, and chronic inflammation [61]. If not managed, type 2 diabetes leads to development of heart disease, stroke, nerve damage, kidney disease, eye disease, and other health complications. The expression levels of NLRP3 inflammasome components are correlated with the disease severity of type 2 diabetes [54]. IL-1β, one downstream product of NLRP3 inflammasome activation, activates the c-Jun N-terminal kinases (JNK) pathway, which promotes insulin resistance by regulating phosphorylation of insulin receptor and insulin receptor substrate-1 [53,62]. IL-1β triggers endoplasmic reticulum stress, which is involved in the pathogenesis of type 2 diabetes [63,64]. IL-1β generated in pancreatic macrophages is a key driver of β-cell death and therefore contributes to pancreatic failure [65].

## 3. Recently Identified Natural Compounds with Anti-NLRP3 Inflammasome Functions

Because of the critical involvement of the NLRP3 inflammasome in a broad range of complex diseases, it has become an attractive therapeutic target. Protein-based therapies that block the action of IL-1*β*, one of its downstream products, have shown promising outcomes in patients with gout, CAPS, and type 2 diabetes in clinical trials [9,66,67], suggesting that inhibition of the NLRP3 inflammasome is likely the right path to curb NLRP3 inflammasome-mediated diseases. However, these protein-based therapies have some inherent limitations. First, their high cost limits their wide application. Second, because these therapeutics are protein-based, they need to be administered by injections, but some patients experience adverse effects such as redness, bruising, swelling or pain at the injection site. Finally, some elegant genetic studies have shown that other downstream products of the NLRP3 inflammasome, such as IL-18 or gasdermin D, contribute to disease progress in animal models [3,27]. Therefore, inhibiting the NLRP3 inflammasome activation instead of targeting its downstream products may offer greater therapeutic promise.

Small chemical molecules are cost-effective and less invasive compared to protein-based therapies. Some synthetic small chemical molecules, such as Bay11-7082 [68], CY-09 [69], and MCC950 [70], have been shown to effectively and directly impede NLRP3 inflammasome assembly and activation. To date, MCC950 is the most potent and specific inhibitor of the NLRP3 inflammasome. Unfortunately, its phase II clinical trial for rheumatoid arthritis was suspended because of hepatic toxicity [71]. For many of these inhibitors, the underlying mechanisms of their inhibitory actions are not clear and need further investigation. Moreover, future studies are warranted to explore their specificity, potency, and long-term safety in humans.

Natural compounds have been a major source of traditional medicines and the source of new drug discovery in history [72,73]. Many blockbuster drugs on the market, such as artemisinin, metformin, and ephedrine, are derived directly or indirectly from plants [72]. In the 20th century, many pharmaceutical companies have shifted focus from isolation and optimization of medicinal compounds from plants or other natural sources to synthesis of chemical libraries because of low reproducibility of crude natural extracts and lack of efficient and rapid strategies to separate and identify the active natural compounds. However, the low success of small-chemical libraries has rekindled the interest of pharmaceutical companies and academic researchers in natural compounds in recent years. Nevertheless, the exceptional structural diversity and highly selective activity of natural products has made, and will continue to make, them versatile and superior sources of drug discovery [72]. The study of natural compounds with anti-NLRP3 inflammasome function is still in its infancy. To date, approximately 40 natural products that inhibit activation of the NLRP3 inflammasome have been identified. An extensive overview of these natural compounds has been provided in two recent reviews published in 2016 and 2017 [10,11]. Here, we provide an update on new natural compounds that have been identified from 2017 to 2020 as inhibitors of the NLRP3 inflammasome.

### 3.1. Oridonin

**Figure 2:**
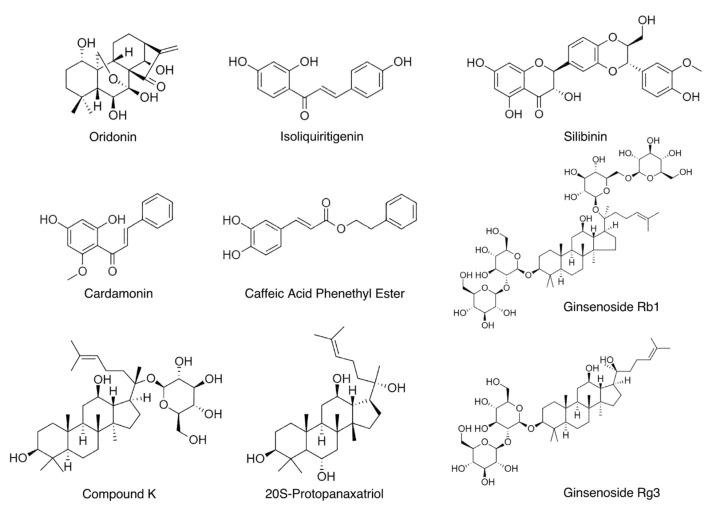
Chemical structures of anti-NLRP3 inflammasome natural compounds.

Oridonin is an ent-kaurane diterpenoid (Figure 2 and Table 1) extracted from *Rabdosia rubescens*, a traditional medicinal herb that has been commonly used to treat inflammatory diseases in China for centuries [74]. Oridonin strongly suppressed NLRP3 inflammasome activation in primary macrophages by blocking formation of the inflammasome complex [75]. This natural compound had no effects on the protein levels of NLRP3, ASC, caspase-1, NEK7, or pro-IL-1β. It also had no impact on potassium efflux or mitochondrial ROS production, when the NLRP3 inflammasome was activated using LPS and nigericin. The inhibitory effects of oridonin on the NLRP3 inflammasome were specific, considering it had no impact on activation of other inflammasomes, including the Absent in melanoma 2 (AIM2) inflammasome and NLR family CARD domain-containing protein 4 (NLRC4) inflammasome. Further investigation revealed that oridonin directly formed a covalent bond with cysteine 279 on the NACHT domain of the NLRP3 protein through a carbon–carbon double-bond. Binding of oridonin to the NLRP3 protein impeded the interaction between NEK7 and NLRP3 and therefore blocked further assembly and formation of the NLRP3 inflammasome complex. In MSU crystal-induced peritonitis and gouty arthritis in mice, oridonin attenuated the NLRP3 inflammasome-dependent acute inflammatory responses. After the mice were fed with a high-fat diet (HFD) for 12 weeks to induce obesity and chronic inflammation, daily intraperitoneal administration of oridonin prevented further body weight gain and improved glucose tolerance and insulin sensitivity [75].

In a recent study [76], oridonin was reported to suppress NLRP3 inflammasome activation in the murine macrophage-like RAW 264.7 cell line. In an LPS-induced acute lung injury mouse model, intraperitoneal administration of oridonin reduced the protein levels of NLRP3, caspase-1, ASC, and IL-1β in lung tissues. Histologically, inflammatory cell infiltration and alveolar hemorrhage of lung were blunted by oridonin treatment. Yan et al. [77] reported that the NLRP3 inflammasome was activated in the pericontusional cerebral cortex upon induction of traumatic brain injury in mice using the weight-drop method. Oridonin reduced both mRNA and protein levels of the *Nlrp3*, *Pycard*, and *Casp1* genes. The levels of IL-1β and IL-18, downstream products of NLRP3 inflammasome activation, were also decreased in the cerebral cortex. Meanwhile, oridonin treatment attenuated the neurological deficits associated with traumatic brain injury.

These mechanistic and pathological studies suggest a high therapeutic potential of oridonin in NLRP3 inflammasome-mediated diseases. Hundreds of oridonin derivatives have been designed and synthesized to improve the potency and bioavailability of oridonin in cancer treatment [74,78]. However, none of these derivatives have been tested for their anti-NLRP3 inflammasome functions. After oral administration of oridonin in Sprague–Dawley rats, 18 oridonin-derived metabolites were detected in bile or urine or both, 17 in bile and 10 in urine [79]. Oridonin mainly underwent reduction, oxidation, dehydroxylation, and glucuronic acid conjugation in vivo. It would be interesting to investigate whether these metabolites demonstrate anti-NLRP3 inflammasome activities.

### 3.2. Isoliquiritigenin

Isoliquiritigenin (ILG, 2′,4′,4-trihydroxychalcone), a flavonoid with a chalcone structure (Figure 2 and Table 1), is the main active compound of roots and rhizomes of Chinese licorice (*Glycyrrhiza uralensis*), which has been used as a natural sweeter and medicinal food worldwide [93]. ILG exhibits various bioactivities, such as antioxidant and anti-inflammatory functions [94]. In 2014, ILG was reported, for the first time, to be a potent inhibitor of the NLRP3 inflammasome [80]. The inhibitory effect of ILG on the NLRP3 inflammasome was considered specific since ILG had no inhibitory effects on the AIM2 inflammasome. It had no impact on mitochondrial ROS production but inhibited ASC oligomerization during NLRP3 inflammasome activation. However, the exact molecular targets of ILG were not further investigated. In the same study, ILG supplement (0.5% *w*/*w*) in the HFD reduced body weight gain (despite similar food intake), improved glucose tolerance and insulin sensitivity, inhibited hepatic steatosis, and suppressed inflammation in epididymal white adipose tissue (eWAT). Expression of the *Nlrp3*, *Casp1*, *Pycard*, and *Il1b* genes was also reduced in the eWAT of ILG-treated mice. Correspondingly, the release of IL-1β and caspase-1 was diminished from ex vivo cultured eWAT of ILG-treated mice [80]. A subsequent study from Lee et al. [81] showed a HFD supplemented with 0.02% (*w*/*w*) ILG did not affect body weight gain but slightly decreased food intake. The discrepancy between these two studies could be attributed to differences in HFD composition and ILG dose. Nevertheless, Lee et al. still found that supplementation of ILG inhibited hepatic steatosis, improved glucose tolerance and insulin sensitivity, and suppressed systemic chronic inflammation [81].

In a rat disease model of intracerebral hemorrhage (ICH), intraperitoneal administration of ILG decreased brain injury and neurological deficits, accompanied by decreased mRNA and protein levels of the *Nlrp3*, *Pycard*, and *Casp1* genes in the ipsilateral hemisphere, as well as reduced levels of mature IL-1β and IL-18 in the brain and blood [82]. In this study, ILG was found to simultaneously dampen NF-κB signaling and boost transcription of the *Nrf2* gene in the damaged brain. NF-κB signaling is involved in the priming step of NLRP3 inflammasome activation [12]. The *Nrf2* gene expresses the transcription factor erythroid-2 related factor 2 (Nrf2), which is a key mediator that controls the expression of many antioxidant and detoxification enzymes and has been implicated in alleviating NLRP3 inflammasome activity [95]. Therefore, the actions of ILG on the priming step and Nrf2 regulation may contribute to its anti-inflammasome function in vivo.

In an LPS-induced acute lung injury mouse model, ILG alleviated the severity of lung injury, reduced the production of IL-1β, and lowered protein levels of NLRP3, ASC, cleaved caspase-1, and pro-IL-1β [83]. In carrageenan-induced pleurisy and lung injury in mice, pre-treatment with ILG reduced lung injury, decreased the levels of cytokines and neutrophil infiltration in the pleural exudate, as well as protein levels of NLRP3, cleaved caspase-1, and mature IL-1β in the lung tissues [84].

ILG has been shown to block NLRP3 inflammasome assembly and therefore suppress activation of the NLRP3 inflammasome in macrophages. ILG also has demonstrated potent anti-inflammatory function in multiple disease models associated with chronic or acute inflammation. Therefore, ILG is a new promising inhibitor of the NLRP3 inflammasome. However, further investigation is warranted to reveal the exact molecular mechanisms of the anti-NLRP3 inflammasome action of ILG.

Four ILG derivatives, including 4,4′-Diacetoxy-2′-hydroxy chalcone, 2′,4′-Dimethoxy-4-hydroxychalcone, 4-Acetoxy-2′,4′-dimethoxychalcone and 2′,4′-Dimethoxychalcone, exhibited potent blood glucose-lowering activities in Swiss albino mice [96]. Another ILG derivative was shown to inhibit cancer cell proliferation [97], and a sixth was found to be neuroprotective [98]. Using human liver microsomes, seven phase I metabolites of ILG—liquiritigenin, 2′,4,4′,5′-tetrahydroxychalcone, sulfuretin, butein, davidigenin, cis-6,4′-dihydroxyaurone, and trans-6,4′-dihydroxyaurone—were identified [99]. In lung cancer cells, ILG was found to be converted into 2, 4, 2′, 4′-Tetrahydroxychalcone (THC) [100]. Interestingly, ILG inhibited activity of kinase Src in these cancer cells but not in the cell-free system, whereas THC inhibited Src activity in both the cancer cells and the cell-free system. These results suggest that THC is the bona fide agent that exerts anti-cancer bioactivity in cancer cells. The effects of these ILG derivatives or metabolites on the NLRP3 inflammasome have not been explored.

### 3.3. Silibinin

Silibinin (SB), also called Silybin, is a natural polyphenolic flavonoid (Figure 2 and Table 1) from milk thistle (*Silybum marianum*), which has been used as an anti-inflammatory and hepatoprotective herb [101]. Incubation of SB with differentiated THP-1 cells (a human monocytic leukemia cell line) for two hours decreased caspase-1 autocleavage when the NLRP3 inflammasome was activated using LPS and ATP [85]. SB had no impact on the protein levels of NLRP3, ASC, and caspase-1 but resulted in decreased ROS production and inflammasome complex formation. Incubation of SB with RAW264.7 cells for three hours inhibited NF-κB signaling. The anti-NLRP3 inflammasome and anti-NF-κB properties of SB may confer its protective effects on LPS-induced acute lung injury in mice [85]. In another study, SB significantly reduced IL-1β release in THP-1 cells when the NLRP3 inflammasome was activated using LPS and MSU [86].

When mice were fed with a HFD to induce liver steatosis, the protein levels of NLRP3 and cleaved caspase-1 in the liver were increased but were largely blocked by the oral administration of SB [87]. In vitro, SB suppressed IL-1β release from primary hepatocytes and HepG2 cells that had been incubated with 100 μM of palmitic acid.

A number of SB derivatives have been synthesized, and many of them have demonstrated increased anti-proliferative potencies, serum stability, and solubility [102,103]. SB mainly underwent hydroxylation in phase I metabolism and glucuronidation in phase II metabolism in vivo [104]. As with other natural compounds, the effects of these SB derivatives and metabolites on the NLRP3 inflammasome have not been investigated.

### 3.4. Cardamonin

Cardamonin (2′,4′-dihydroxy-6′-methoxychalcone, CDN) is the major flavonoid (Figure 2 and Table 1) extracted from the seeds of *Alpinia Katsumadai*, a traditional Chinese medicinal herb of the ginger family that has been used for the treatment of multiple inflammatory disorders [105]. This natural compound was found to suppress caspase-1 autocleavage and IL-1β release upon NLRP3 inflammasome activation in immortalized mouse bone marrow-derived macrophages (BMDMs), primary BMDMs, human THP-1 cell line-differentiated macrophages, and human PBMCs [88]. It is interesting to note that CDN was incubated with cells for one hour after the LPS priming step but before the addition of various NLRP3 activators, suggesting that the action of CDN on NLRP3 inflammasome activity was direct and prompt. The inhibitory effects of CDN on the NLRP3 inflammasome were specific because it did not inhibit activation of the NLRC4 or AIM2 inflammasome. One-hour incubation of CDN did not affect the protein levels of NLRP3, ASC, or caspase-1 but impeded the assembly of the NLRP3 inflammasome. When CDN was intraperitoneally injected one hour prior to LPS injection, CDN protected mice from death and significantly reduced serum IL-1β levels. The in vivo efficacy of CDN was comparable to MCC950 [88], which is a well-established potent chemical inhibitor of the NLRP3 inflammasome [70].

In another study [89], similar doses of CDN were incubated with THP-1 cells or BMDMs for 21 h, followed by NLRP3 inflammasome activation. The lengthy incubation of cells with CDN decreased the NLRP3 protein level and expression of the *Il1b* gene, accompanied by decreased levels of cleaved caspase-1 and mature IL-1β. Further investigation indicated that the aryl hydrocarbon receptor (AhR) and its downstream axis Nrf2/NADPH:quinon acceptor oxidoreuctase 1 (NQO1) mediated the inhibitory effects of CDN on NLRP3 inflammasome activity [89], although the study did not further elucidate whether AhR/Nrf2/NQO1 targeted the priming step or activating step during NLRP3 inflammasome activation. The same study showed that oral or rectal administration of CDN significantly alleviated disease severity and reduced inflammation in dextran sulfate sodium (DSS)- or 2,4,6-trinitrobenzene sulfonic acid (TNBS)-induced colitis in mice, suggesting the promising therapeutic potential of CDN in the management of inflammatory bowel disease.

Nineteen derivatives of CDN were synthesized, and some of them showed increased apoptosis-inducing potencies in cancer cell lines [106]. A recent study [107] identified 40 metabolites of CDN after it was orally administered to rats. These metabolites were mainly derived from methylation, demethylation, hydrogenation, hydroxylation, dehydroxylation, glucuronidation, and sulfation of CDN in vivo. It would be important to determine the effects of CDN derivatives or metabolites on the NLRP3 inflammasome in future studies.

### 3.5. Caffeic Acid Phenethyl Ester

Caffeic Acid Phenethyl Ester (CAPE), a natural polyphenolic compound (Figure 2 and Table 1) in honeybee propolis, has been reported to possess antioxidant and anti-inflammatory properties [108]. Remarkably, CAPE suppressed caspase-1 autocleavage, IL-1β release, and ASC oligomerization when LPS-primed BMDMs were treated with MSU to activate the NLRP3 inflammasome [90]. In MSU-induced gouty arthritis in mice, orally administered CAPE significantly attenuated foot swelling and neutrophil infiltration in foot tissues, accompanied by decreased release of IL-1β and IL-18 and reduced levels of cleaved caspase-1 in foot tissues. Further mechanistic studies using reconstitution experiments, pull down assay, and surface plasmon resonance (SPR) analysis showed that CAPE directly bound to ASC. CAPE specifically bound to PYD of ASC, but not to PYD of NLRP3 or CARD of ASC. Molecular modeling analysis suggested that CAPE seemed to interact with Glu13 and Lys24 of ASC through hydrogen bonds and with Lys21 and Leu45 through lipophilic interactions. The association of CAPE with ASC prevented the interaction of ASC and NLRP3 in BMDMs upon inflammasome activation. Because CAPE targeted ASC, the AIM2 inflammasome, which is composed of the sensor AIM2, the adaptor ASC, and the effector caspase-1, was unsurprisingly inhibited by CAPE treatment [90].

A recent study [91] showed that CAPE suppressed caspase-1 autocleavage, IL-1β release, and inflammasome formation in both THP-1 cells and BMDMs when the NLRP3 inflammasome was activated using LPS and ATP. The level of NLRP3 protein was decreased in a dose-dependent manner by CAPE treatment. Interestingly, CAPE did not affect the mRNA level of the *Nlrp3* gene. Instead, during NLRP3 inflammasome activation, CAPE suppressed deubiquitination of the NLRP3 protein, which is a prerequisite for the assembly of the NLRP3 inflammasome complex [5]. CAPE was found to promote interaction of NLRP3 with the ubiquitin-conjugating enzyme Cullin1 but to decrease interaction of NLRP3 with the deubiquitinating enzyme COP9 signalosome subunit 5 (CSN5). This study suggests another mechanism by which CAPE suppresses activation of the NLRP3 inflammasome. CAPE was also shown to suppress NLRP3 inflammasome activity in human aortic valve interstitial cells (AVICs) cultured in osteogenic induction medium [92].

Over 100 derivatives of CAPE were synthesized and most of them were tested for their anti-cancer properties [109]. Recently, 48 CAPE derivatives were designed, synthesized, and tested for their anti-inflammatory properties [110]. Although CAPE mildly inhibited secretion of tumor necrosis factor alpha (TNFα) in LPS-treated mouse peritoneal macrophages, in the initial screen, one of its derivatives, 10 s, potently inhibited the level of TNFα in the medium. This 10s derivative was found to bind to myeloid differentiation protein 2 (MD2) with high affinity through two key hydrogen bonds and hydrophobic interactions. The association of 10s and MD2 blocked the interaction of LPS and MD2 and thus blunted activation of the NF-κB pathway. Therefore, in the context of NLRP3 inflammasome activation, 10s should strongly inhibit the priming step. It would be very interesting to assess whether 10s retains the ability to bind to ASC and/or mediate NLRP3 deubiquitination.

When CAPE was orally given to rats, the level of caffeic acid was remarkably enhanced in urine collected within 24 h, along with moderate increase in caffeic acid phenethyl ester, caffeic acid ethyl ester, and caffeic acid 2-(2-ethoxyethoxy)ethyl ester [111]. Future studies are needed to investigate anti-NLRP3 inflammasome functions of these CAPE metabolites.

### 3.6. Ginsenosides

Ginsenosides are steroid glycosides and triterpene saponins (Figure 2 and Table 2) that are specifically enriched in the ginseng, the root of plants in the genus *Panax*. Their herb medicines have been used in Asia for thousands of years to improve stamina and treat a variety of diseases, such as gastric ulcer, diabetes, and cancer [112]. More than thirty ginsenosides have been identified, among which Rb1, compound K (CK), Rg3, and 20S-protopanaxatriol (PPT) have been indicated in inhibiting NLRP3 inflammasome activity [113].

Rb1 is the most abundant ginsenoside in Korean ginseng (*Panax ginseng*), and CK is a metabolite of Rb1 generated through hydrolysis by intestinal bacteria [119]. Chen et al. [114] observed when eWAT of mice were cultured ex vivo with high glucose (33 mM) for 24 h, the level of NLRP3 protein and cleaved caspase-1 in the eWAT tissue and IL-1β release in the medium were increased, but all were blunted by pretreatment with Rb1 or CK. In diabetes spontaneous mutation (*Lepr^db^*) mice (often called db/db mice), oral administration of CK improved glucose tolerance, insulin sensitivity, cognitive function, and memory [115]. Remarkably, the levels of mature IL-1β, NLRP3, cleaved caspase-1, and ASC were decreased in the hippocampus of db/db mice treated with CK. Although these animal studies pointed to the possible regulatory effects of Rb1 and CK on the NLRP3 inflammasome, the experimental design was not able to determine whether the decreased IL-1β release and cleaved caspase-1 in the tissues were caused by decreased levels of the NLRP3 inflammasome subunits or due to diminished activation of the NLRP3 inflammasome. The direct effects of Rb1 or CK on NLRP3 inflammasome activation need to be further investigated.

In a recent study [116], ginsenoside Rg3 was shown to specifically inhibit activation of the NLRP3 inflammasome, but not the NLRC4 or AIM2 inflammasome. In LPS-primed THP-1 cells, Rg3 reduced caspase-1 autocleavage and the secretion of IL-1β and IL18, but it had no impact on the release of IL-6 or TNFα when the NLRP3 inflammasome was stimulated by nigericin, ATP, or alum. Rg3 treatment did not affect upstream events of NLRP3 inflammasome activation, such as mitochondrial ROS production or potassium efflux. Instead, Rg3 abrogated the interaction of NEK7 and NLRP3 and therefore disrupted NLRP3 inflammasome assembly. In LPS-induced endotoxic shock in mice, pretreatment with Rg3 via intraperitoneal injection suppressed the levels of IL-1β in both the peritoneal cavity and circulation and improved the survival of mice [116]. Rg3 was metabolized into six metabolites after its oral administration in rats [120]. Therefore, it is important to determine whether Rg3 could reach innate immune cells in vivo and/or whether any Rg3 metabolites retain inhibitory effects on the NLRP3 inflammasome.

Ginsenoside PPT is one of the terminal metabolites commonly detected in human circulation after ingestion of ginseng [121]. Jiang et al. reported that PPT suppressed activation of the NLRP3 inflammasome, but not AIM2 or NLRC4 inflammasome, in LPS-primed peritoneal macrophages [117]. PPT blocked ASC oligomerization and subsequent caspase-1 autocleavage and IL-1β release upon NLRP3 inflammasome activation by nigericin, ATP, or silica, but it had no impact on the protein levels of NLRP3, ASC, or caspase-1. In LPS-induced endotoxic shock in mice, PPT decreased serum IL-1β levels. In the mouse model of MSU-induced acute peritonitis, PPT attenuated the level of IL-1β and neutrophil infiltration in peritoneal lavage fluids [117]. In another recent study, PPT was shown to ameliorate thioacetamide-induced hepatic fibrosis and decrease protein levels of NLRP3, cleaved caspaspe-1, and mature IL-1β in fibrotic livers [118].

A number of ginsenoside derivatives have been reported [122,123] and their functional significance in inhibition of the NLRP3 inflammasome needs further study.

## 4. Conclusions and Perspectives

Tozser et al. in 2016 [11] summarized nine natural compounds with anti-NLRP3 inflammasome function. Most of these natural compounds were included in a more recent review by Jahan et al. in 2017 [10], which overviewed 34 natural compounds possessing inhibitory actions on the NLRP3 inflammasome. In the last three years, five more new natural compounds have been identified with anti-NLRP3 inflammasome properties; these are summarized in this review. Although ILG was included in Jahan’s review, only one report was available at that time. Therefore, we added ILG to the list of most recently identified natural compounds and included an overview of ILG’s actions based on four more recent research articles.

The study of natural compounds with anti-NLRP3 inflammasome function is still at an early stage. Many outstanding questions remain unanswered in this new field. Although some elegant studies have shown that oridonin or CAPE inhibits activation of the NLRP3 inflammasome through unique mechanisms or by targeting multiple targets, more investigation is warranted to elucidate the detailed molecular mechanisms that enable each natural compound to inhibit the NLRP3 inflammasome. It also would be important to study the structural basis of anti-inflammasome properties of these natural compounds and their derivatives, since such studies will facilitate modification and optimization during drug development. Future studies on the bioavailability, efficacy, and safety of these natural compounds in vivo are needed, as such information is critical to determine their suitability for treatment of specific diseases. In addition, because natural compounds could be converted into a variety of metabolites after oral administration, it would be critical to assess what types of metabolites reach innate immune cells and whether these metabolites retain anti-NLRP3 inflammasome activities. An ultimate question in the field of anti-NLRP3 inflammasome natural compounds is how to develop efficient and rapid strategies to fractionate and separate the crude natural extracts and identify new active natural components. The whole natural compound field is facing the same challenge, requiring multidisciplinary efforts and expertise from chemistry, molecular and cell biology, physiology, and pharmacology.

Successful development of a drug from bench to bedside requires tremendous financial and time investment. Generally, it takes 10–15 years and USD 500 million–USD 2 billion to develop a single drug [124,125]. Promising drug leads undergo comprehensive examination in preclinical studies of absorption, distribution, metabolism, and excretion (ADME), efficacy, and toxicity, followed by modification and production optimization. Afterward, the candidates selected from preclinical studies enter the clinical trials (typically four phases) and are evaluated for their efficacy and safety in humans. Unfortunately, the whole process is associated with high failure frequency often due to low efficacy in humans [126]. For example, only 5% of oncology drugs entering Phase I clinical trials are eventually approved by the US Food and Drug Administration (FDA) [127]. Compared to synthetic chemicals, development of natural product-sourced drugs is more time consuming and associated with higher cost, due to the extra efforts needed to identify the active components in crude extracts and to modify (often extensively) the base molecules [72]. Therefore, there is a long way to go for natural products with anti-NLRP3 inflammasome functions to be eventually developed into drugs.

When these natural compound-based drugs become available in clinic, their cost-effectiveness would facilitate their routine use in treatment of chronic diseases such as autoinflammatory disease CAPS. Very often, such natural compound-derived drugs can be taken orally and thus avoid the side effects associated with frequent injection. However, one complication of using NLRP3 inflammasome inhibitors in treating chronic inflammation lies in potential inherent adverse effects. Proper activation of the NLRP3 inflammasome is part of the host’s protective inflammatory response against pathogens and other detrimental insults [2,128]. Long-term and continuous inhibition of the NLRP3 inflammasome may compromise the host’s normal immune defense function and therefore increase the risk of infections. One possible solution is to use a highly specific drug against the NLRP3 inflammasome and thus leave other innate immune responses intact. Intermittent administration of these inhibitor-based drugs could be another option. Furthermore, selection of different natural compounds with different molecular targets or tissue distribution could help to reach maximal clinical outcomes while limiting undesirable side effects.

Finally, it is worthwhile mentioning that many natural compounds could function through highly selective and totally unexpected new pathways. Therefore, investigating the molecular mechanisms underlying their anti-NLRP3 inflammasome functions could possibly reveal new insights and pathways of NLRP3 inflammasome activation, thus contributing to a better understanding of the complex mechanisms of NLRP3 inflammasome activation.

## Figures and Tables

**Table 1:** Natural compounds with anti-NLRP3 inflammasome functions.

Name (Natural Source)	In Vitro Model and Dose	Animal Model and Dose	Disease Model	Inhibitory Mechanism	References
Oridonin (*Rabdosia rubescens*)	BMDMs, human PBMCs: 0.5–2 μM	WT and *Nlrp3*^−/^^−^ C57BL/6J mice: 3–20 mg/kg	Peritonitis, Gouty arthritis, HFD-induced obesity	Bound to cysteine 279 of the NLRP3 protein, impeded interaction between NEK7 and NLRP3 and thus blocked NLRP3 inflammasome assembly	He [75]
	RAW 264.7: 2.5–10 μM	C57BL/6 mice: 20–40 mg/kg	Acute lung injury	Reduced protein levels of NLRP3, ASC, caspase-1, and IL-1β in lung	Yang [76]
		C57BL/6 mice: 10 mg/kg	Traumatic brain injury	Reduced both mRNA and protein levels of the *Nlrp3*, *Pycard*, *Casp1* genes in cerebral cortex	Yan [77]
Isoliquiritigenin (*Glycyrrhiza uralensis*)	BMDMs, THP-1: 1–10 μM	C57BL/6 mice: 0.5% *w*/*w* in the diet	HFD-induced obesity	Blocked assembly of the NLRP3 inflammasome; reduced expression of the *Nlrp3*, *Pycard*, *Casp1*, and *Il1b* genes in eWAT	Honda [80]
		C57BL/6J mice: 0.02% *w*/*w* in the diet	HFD-induced obesity		Lee [81]
		Sprague-Dawley rats: 10–40 mg/kg	Intracerebral hemorrhage	Reduced both mRNA and protein levels of the *Nlrp3*, *Pycard*, *Casp1* in ipsilateral hemisphere; induced transcription of the *Nrf2* gene; suppressed NF-κB activation	Zeng [82]
	RAW 264.7: 20 μM	C57BL/6J mice: 30 mg/kg	Acute lung injury	Reduced protein levels of NLRP3, ASC, cleaved caspase-1, and pro-IL-1β in lung	Liu [83]
		BALB/c mice: 30 mg/kg	Pleurisy and lung injury	Reduced protein levels of NLRP3, cleaved caspase-1, and mature IL-1β in lung	Gao [84]
Silibinin (*Silybum marianum*)	THP-1, RAW 264.7: 25–100 μM	C57BL/6 mice: 50–100 mg/kg	Acute lung injury	Reduced ROS production; blocked assembly of NLRP3 inflammasome; suppressed NF-κB activation	Zhang [85]
	THP-1, human PBMCs: 50 μM			Reduced transcription of the *NLPR3*, *CASP1*, and *IL1B* genes	Matias [86]
	HepG2, primary hepatocytes: 0.2–50 μM	C57BL/6 mice: 50–100 mg/kg	HFD-induced liver steatosis	Reduced protein levels of NLRP3 and cleaved caspase-1 in liver	Zhang [87]
Cardamonin (*Alpinia Katsumadai*)	BMDMs, iBMDMs, THP-1, human PBMCs: 1–20 μM	C57BL/6 mice: 25–50 mg/kg	Endotoxic shock	Blocked assembly of the NLRP3 inflammasome	Wang [88]
	BMDMs, THP-1: 1–30 μM	C57BL/6 mice, BALB/c mice: 15–60 mg/kg	Colitis	Reduced NLRP3 protein level and transcript of the *Il1b* gene; activated AhR/Nrf2/NQO1 pathway	Wang [89]
Caffeic acid phenethyl ester (Honeybee propolis)	BMDMs: 0.5–10 μM	WT and *Nlrp3*^−/^^−^ C57BL/6 mice: 30 mg/kg	Gouty arthritis	Bound to ASC, abrogated interaction between ASC and NLRP3, and therefore blocked NLRP3 inflammasome assembly	Lee [90]
	BMDMs, THP-1: 5–20 μM	C57BL/6 mice: 5–45 mg/kg	Colitis-associated cancer	Reduced NLRP3 protein level, but had no impact on transcription of the *Nlrp3* gene; suppressed deubiquitination of the NLRP3 protein through enhancing the interaction between NLRP3 and Cullin1 and decreasing the interaction between NLRP3 and CSN5	Dai [91]
	Human aortic valve interstitial cells: 10 μM			Reduced protein levels of NLRP3 and ASC; inhibited NF-κB pathway	Liu [92]

Abbreviations: BMDMs: bone marrow-derived macrophages; PBMCs: peripheral blood mononuclear cells; wt: wildtype; HFD: high-fat diet; eWAT: epididymal white adipose tissue; ROS: reactive oxygen species; iBMDMs: immortalized BMDMs.

**Table 2:** Ginsenosides with anti-NLRP3 inflammasome functions.

Name of Ginsenosides	In Vitro Model and Dose	Animal Model and Dose	Disease Model	Inhibitory Mechanism	Refe-rences
Ginsenoside Rb1	eWAT: 10 μM			Reduced protein levels of NLRP3 and cleaved caspase-1 in eWAT cultured ex vivo	Chen [114]
Compound K		db/db mice: 10 mg/kg	Diabetes	Reduced protein levels of NLRP3, ASC, cleaved caspase-1, and IL-1β in hippocampus	Li [115]
	eWAT: 10 μM			Reduced protein levels of NLRP3 and cleaved caspase1 in eWAT cultured ex vivo	Chen [114]
Ginsenoside Rg3	THP-1, RAW 264.7, BMDMs: 1–10 μg/mL	C57BL/6J mice: 10 mg/kg	Endotoxic shock	Impaired the interaction of NEK7 and NLRP3 and therefore blocked assembly of the NLRP3 inflammasome	Shi [116]
20S-protopanax-atriol	Mouse peritoneal macrophages: 10–40 μM	C57BL/6 mice: 5–10 mg/kg	Peritonitis, Endotoxic shock	Blocked assembly of the NLRP3 inflammasome	Jiang [117]
	HSC-T6: 10–20 μM	C57BL/6J mice: 10–20 mg/kg	Hepatic fibrosis	Reduced protein levels of NLRP3, cleaved caspase-1, and mature IL-1β in fibrotic liver	Song [118]

Abbreviations: eWAT: epididymal white adipose tissue; BMDMs: bone marrow-derived macrophages.

## Data Availability

Not applicable.
